# NeuroBoricuas: A Culturally Rooted Approach to Neuroscience Outreach and Research Training

**DOI:** 10.1523/ENEURO.0334-25.2025

**Published:** 2025-10-17

**Authors:** Christian Bravo-Rivera

**Affiliations:** Departments of Psychiatry and Anatomy and Neurobiology, University of Puerto Rico School of Medicine, San Juan, Puerto Rico 00936

**Keywords:** community outreach, cultural identity, neuroscience education, peer teaching, Puerto Rico, student training pipeline

## Abstract

Puerto Rico's cultural identity, shaped by Taíno heritage, Spanish colonization, and US governance, emphasizes family, music, food, and collective participation. Drawing on these traditions, we developed NeuroBoricuas, a grassroots neuroscience education movement that reimagines outreach through cultural metaphors of parrandas and peer teaching. What began as a reflection on conventional Brain Awareness Week evolved into student-led demonstrations, the creation of the first neuroscience laboratory in a Puerto Rican high school, and a network of university chapters and high school clubs across the Island. These groups lead workshops, classroom visits, and community events, positioning students as both learners and teachers of neuroscience. A partnership with Backyard Brains provided affordable, hands-on tools that made neuroscience tangible for K-12 and university students, while events such as Explora tu Cerebro en la SanSe integrated science into Puerto Rico's most iconic cultural festival. To extend beyond outreach, the Bravo Lab Immersive Summer Program (BLISP) was launched in 2022, immersing undergraduates in advanced approaches including optogenetics, fiber photometry, and behavioral assays. BLISP emphasizes mentoring and peer-to-peer training, building confidence and scientific identity while creating pathways to graduate training. Recent collaborations, including NeuroBridges with the University of California, Irvine, have further expanded opportunities for Puerto Rican students through international partnerships. Together, NeuroBoricuas and BLISP demonstrate a scalable model of science education rooted in culture, identity, and community. Like a parranda, this movement grows as new voices join in, showing that neuroscience thrives when it is shared, celebrated, and carried forward collectively.

## Introduction

Puerto Rico is a Caribbean island with a very singular history. Long before the Spanish conquest of the sixteenth century, the Island was called *Borikén* by its Taíno inhabitants (Figueroa-Mercado, 1996). From Borikén comes the word *Boricua*, the name Puerto Ricans proudly use to identify ourselves as descendants of the Island's first people. By the end of the nineteenth century, the United States had invaded the Island, then widely known as Puerto Rico. Since then, our identity has been defined by a fusion: a deeply rooted Latin American cultural heritage shaped alongside heavy US influence, given our colonial status (Alegría, 1999). Today, the Island remains under US federal oversight, yet we conserve core Latin American traditions such as our emphasis on family and community, our love for music and dance, our passion for sports, our devotion to food, and our high regard for health care and education.

Our celebrations embody this fusion. Christmas celebrations begin even before Christmas Eve and extend all the way to Three Kings Day in January. These weeks are filled with folkloric music, food, and late-night gatherings. At the center of the season are parrandas, when groups of friends show up unannounced at night, armed with our musical instruments *panderos*, *güiros*, and the *cuatro*. They sing a traditional songlist, familiar verses and melodies passed down through generations that everyone joins in without needing sheet music. Hosts welcome them with food such as *lechón asado*, *arroz con gandules*, and *pasteles*, and the celebration continues until dawn. Parrandas are not just music or parties; they are a living tradition where cultural memory is carried forward by collective participation (Gleason, 2003). I was born and raised in this environment, where joy, knowledge, and identity were inseparable, and for a long time I knew no other way of life but our own.

When I joined Gregory Quirk's lab as an undergraduate and later continued into my doctoral training at the University of Puerto Rico (UPR) School of Medicine, I found that this cultural backdrop shaped our lab as well. My labmates became brothers and sisters, and Greg was very much a father figure. Although Greg welcomed postdocs from around the world, they too embraced our family-style lab culture. We did not compete with one another; instead, we celebrated each other's successes as our own and lifted one another through the challenges of science. Only when I experienced laboratories outside Puerto Rico did I realize how rare this was. What I had taken for granted was not simply a research environment but an expression of our Puerto Rican identity fused with our scientific endeavors.

Greg also emphasized the importance of sharing our research beyond academia. Each year he encouraged us to participate in Brain Awareness Week (BAW), bringing science to local schools. During my final year as a PhD student, he gave me the chance to choose the high school for our BAW visit. I chose my alma mater, CROEM. Some labmates rolled their eyes since it was nearly a 3 h drive across the Island. On the way there, a labmate joked, “Thank God you’re leaving so we don't have to do this long road trip anymore.” The comment struck me. Why not train the high school students themselves to take ownership of BAW, rather than parachuting in for a single day of demos? Since the lab won't visit again, let's make it so that they don't need us to visit anymore.

That question stayed with me because it echoed the way I had grown up. In Puerto Rico, traditions such as food, music, and parrandas are not handed down as lectures but as lived experiences where everyone takes part and contributes. The parranda works because the songs are known by heart, sung together, and carried forward from one generation to the next. Knowledge is alive when it is shared in community.

Out of this reflection, NeuroBoricuas was born. Its very name connects science to heritage. Just as Boricua comes from Borikén, linking us to our Taíno roots, NeuroBoricuas ties neuroscience to that same lineage of cultural identity. Its core idea is peer teaching as empowerment. Instead of presenting neuroscience as something delivered by distant experts, we equip local students to become teachers and ambassadors within their own schools and neighborhoods. In this way, neuroscience knowledge is passed down like parranda traditions: learned in community, carried forward with pride, and made stronger with every new voice that joins in.

## The First Neuroscience Lab in a Puerto Rican High School

The idea of peer teaching quickly evolved into something more ambitious. In 2017, I visited Puerto Rico from my postdoc at New York, and together with the first NeuroBoricuas, we inaugurated the first neuroscience laboratory in a Puerto Rican high school at CROEM. This was more than symbolic; it gave students a physical space, with microscopes, electrophysiology setups, and models, where they could explore neuroscience firsthand. All the equipment was crowd-funded. Press coverage from outlets like *El Nuevo Día* (El Nuevo Dia, 2017) and *Primera Hora* (Primera Hora, 2017) amplified the achievement, showing the Island that neuroscience could take root in public schools.

The lab at CROEM became a nucleus. Students didn't just absorb facts about neurons and synapses; they built demonstrations, led tours, and taught younger classmates. Over time, they became the face of neuroscience in their own community. That peer-led model spread as other schools and university campuses adopted the approach, creating a ripple effect that no single outreach talk could ever achieve.

## Partnering with Backyard Brains

From the beginning, we knew NeuroBoricuas had to be more than lectures. If students were to embrace neuroscience as their own, they needed to experiment and see the brain in action. Our partnership with Backyard Brains (BYB) made that possible.

Founded by neuroscientists Tim Marzullo and Greg Gage, BYB's mission is to make neuroscience accessible. Their low-cost, open-source equipment allows students of all ages to explore the nervous system in real time. Through this collaboration, our schools acquired affordable neuroscience kits that opened a window into the brain.

With these tools, students record muscle signals, control a mechanical claw, or even send one student's neural signals to move another's arm. Many assemble the kits themselves, learning how science and engineering fit together before running experiments.

The kits became the instruments of our scientific parranda. Just as a parranda comes alive when someone brings a *pandero*, another a *güiro*, and another a *cuatro*, these devices let each student contribute to the rhythm of discovery. Some record signals, others code or explain results, and together they create a song of curiosity greater than any one participant.

This partnership has been crucial for making science tangible. Instead of passively receiving information, students become experimenters. Teachers integrate neuroscience into their courses, and students pursue independent projects. In Puerto Rico, where science resources are often scarce, BYB has shown young people that science is not distant but something they can do, build, and share, like a parranda of knowledge carried forward by all who join in.

## Genesis of NeuroBoricuas University Chapters and High School Clubs

By the time NeuroBoricuas matured, it was clear the initiative needed a structure that reached beyond individual schools. The enthusiasm of undergraduates and graduate students across Puerto Rico led to the creation of university chapters. These became like new ensembles in the parranda of NeuroBoricuas, each adding its own rhythm and energy.

Chapters meet regularly, recruit peers, and carry the mission of neuroscience literacy forward. Today they are present across UPR campuses—Río Piedras, Mayagüez, Bayamón, Arecibo, Cayey, Ponce, and the Medical Sciences Campus—as well as at Carlos Albizu University, the Pontificia Universidad Católica de Puerto Rico in Ponce, and Ponce Health Sciences University. These groups host outreach events, teacher workshops, and lab visits and represent NeuroBoricuas at festivals and conferences. Like a parranda moving from house to house, they spread the rhythm of science across the Island.

Alongside these chapters, NeuroBoricuas Clubs have flourished in high schools. The earliest began at CROEM in Mayagüez, soon followed by CROEV in Villalba, CIMATEC in Caguas, TASIS in Dorado, and University Gardens High School in San Juan. Students organize outreach events, run journal clubs, and even train their teachers to use neuroscience equipment. They also use the kits to perform scientific fair projects and have represented Puerto Rico at international competitions, such as in the International Science and Engineering Fair. Just as young voices learn the verses of a parranda and sing them proudly, these students take up the instruments of science and carry its rhythm into their own communities.

Chapter and club members also unite for *Explora tu Cerebro en la SanSe*, launched by Manuel Díaz Ríos and Demetrio Sierra Mercado, among the first NeuroBoricuas. This event fuses neuroscience education with Puerto Rico's most iconic cultural festival, the *Fiestas de la Calle San Sebastián*. In the same streets where parrandas fill days and nights, NeuroBoricuas set up stations where families explore brain function and record neural signals. Explora tu Cerebro turns SanSe into a stage where science and culture meet, showing that the rhythm of the Island can embrace the rhythm of the brain.

The first large-scale workshop, where over a hundred university volunteers became NeuroBoricuas, set the tone for what these chapters and clubs could accomplish. From there, the movement spread across campuses and schools, each one extending the rhythm of curiosity to a wider circle. Together they show that science, like the parranda, thrives when it is shared, taught, and celebrated in community.

## From Outreach to Pipeline: the Bravo Lab Immersive Summer Program (BLISP)

In 2021, I started my lab at the UPR School of Medicine, taking over the space and legacy of my mentor Greg Quirk as he moved to the Philippines to develop behavioral neuroscience research there. At that moment, we felt a surge of enthusiasm for neuroscience across the Island. Yet research opportunities outside San Juan and Ponce remained scarce. Students inspired by NeuroBoricuas wanted to continue, but the path toward advanced training was often blocked by geography and resources.

To help close this gap, we launched the BLISP in 2022 as a NeuroBoricuas initiative. If NeuroBoricuas lit the spark of curiosity, BLISP was designed to carry it into deeper training. The program offers undergraduates a window into the daily life of a neuroscience lab, exposing them to approaches such as optogenetics, fiber photometry, intracranial surgeries, and behavioral assays. Unlike many internships, BLISP has no dedicated grant. Fellows join our lab simply to share in our work, learning side by side with us as we move projects forward.

What makes BLISP distinct is its immersion and mentoring. Fellows are treated not as visitors but as members of the lab family. They attend meetings, contribute to journal clubs, and analyze data. As one student reflected, “I came in expecting to just observe, but instead I felt like part of the team from day one. BLISP didn't just show me neuroscience, it showed me that I belong in neuroscience.” Another wrote, “I never thought I would be trusted to handle real experiments as an undergrad. BLISP gave me that confidence.”

Demand quickly outpaced our capacity. Last year, less than 10% of applicants could be accepted. To respond, we added a virtual component, opening demonstrations and meetings on Zoom. Virtual fellows can now watch experiments, join discussions, and analyze shared datasets. While nothing replaces hands-on work, this track has allowed hundreds more students across the Island to access mentorship and training.

The impact is already visible. Several alumni have gone on to graduate school and medical school, and some have joined my own lab for further training. Their success shows how powerful a short but intensive summer can be when it combines technical exposure with strong mentoring. One former fellow summarized it best: “Before BLISP, I was interested in science. After BLISP, I knew I wanted to dedicate my life to research.”

BLISP is short in duration but long in impact. Fellows shadow surgeries, observe experiments, collect data, and present analyses. They leave with confidence, skills, and a vision of themselves as scientists. Like NeuroBoricuas, BLISP reflects Puerto Rican cultural values of community and peer-to-peer learning. A fellow who masters a task 1 week often trains others the next, multiplying training capacity in a resource-limited setting. This rhythm of shared responsibility mirrors the parranda: what begins with a few voices quickly grows into something collective and sustaining.

Together, NeuroBoricuas and BLISP form a pipeline for Puerto Rican talent, one that begins with a student strumming a cuatro in a classroom demonstration and continues all the way to advanced neuroscience research. By linking outreach and training, we are building not just individual careers but an Island-wide community of future scientists.

## Scalability: Challenges and Opportunities

The success of NeuroBoricuas and BLISP shows what is possible when science is taught through identity, mentorship, and immersion. Yet scaling this model requires careful attention to context. Puerto Rico is a US territory with limited research infrastructure and systemic educational inequities but also with a resilient and culturally vibrant community. Some principles are universal; others must be adapted to local realities.

One transferable principle is peer teaching. NeuroBoricuas thrives because students are not passive recipients of information but active teachers and leaders. This train-the-trainer model can be applied anywhere where resources are scarce, empowering local students to become ambassadors in their own schools and communities. Knowledge then spreads not through parachuting experts but through organic community growth.

Immersive lab training is another scalable feature. While not every institution can replicate optogenetics and photometry, the key is to provide authentic, hands-on science. In resource-limited settings this might mean behavioral experiments, virtual simulations, or partnerships with nearby universities. What matters is giving students a real sense of what scientists do.

Cultural grounding, however, cannot be transplanted wholesale. NeuroBoricuas resonates because it is rooted in Puerto Rican identity, from the use of Spanish to the pride embedded in the word Boricua. A similar program in Appalachia might emphasize community resilience, while in an urban US context it might link science to mental health or social justice. The model works not by exporting Puerto Rican culture but by anchoring science in whatever local idiom gives students pride and belonging.

Logistical growth depends on partnerships and funding. NeuroBoricuas has benefitted from collaborations with the UPR, professional societies, and foundations. Elsewhere, similar initiatives would need to cultivate alliances with local universities, nonprofits, and industries. Government programs and philanthropic science education grants offer concrete paths to support. Scalability hinges not only on program design but on institutional commitment and investment.

## Broader Significance: A Model for Underserved Communities

At its heart, NeuroBoricuas is more than an educational program; it is a social intervention. It intersects science, equity, and cultural identity in ways that conventional outreach often overlooks.

It redefines who gets to do science. By bringing neuroscience into public schools and training Puerto Rican undergraduates in advanced methods, the program challenges the notion that cutting-edge research belongs only to elite mainland universities. It shows that excellence can emerge from marginalized contexts when students are given mentorship, access, and validation.

It also creates a feedback loop between science and community. Fellows do not simply learn photometry or optogenetics; they reflect on how these tools connect to Puerto Rico's realities, from health disparities to environmental stressors. This grounding ensures the next generation of neuroscientists is not only technically skilled but also socially responsive.

Finally, it offers a flexible framework other underserved regions can adopt. Early exposure through outreach, immersive training through BLISP, and recursive mentorship as alumni return to guide new students create a sustainable pipeline. The scientific content may vary, but the logic remains: meet students where they are, empower them as leaders, and create pathways to higher engagement.

## Closing Reflections

In 2023, I was honored to receive the Society for Neuroscience Science Educator Award, the most meaningful recognition of my career. However, the award does not belong to me alone. It belongs to every NeuroBoricua who has passionately learned and taught neuroscience with love and care. Standing on that stage, in front of the largest neuroscience community in the world, I knew our NeuroBoricuas felt that the world was watching them, following their lead in the passionate way they transmit knowledge. What began as a spark in Puerto Rico has become an example for others far beyond our Island.

The parranda has always been our metaphor, and it remains the clearest way to capture this movement. In Puerto Rico, a parranda begins with a few instruments and voices but grows as more people join in, until the whole community sings together. NeuroBoricuas began in the same way, with a small group of students daring to teach and learn science as their own. Now the song has spread through classrooms, universities, festivals, and laboratories. It is a rhythm of belonging, discovery, and joy that cannot be confined to one Island alone.

As NeuroBoricuas continue to grow, they show that science is not only something to be studied but something to be lived and shared. The rest of the world is welcome to join in our parranda of neuroscience, adding new verses and rhythms and carrying the song into their own communities. What started in Puerto Rico can become a global tradition, proving that science, like music, is at its most powerful when it is collective, cultural, and full of heart.

## Synthesis

Reviewing Editor: Mihaela Iordanova, Concordia University - Loyola Campus Decisions are customarily a result of the Reviewing Editor and the peer reviewers coming together and discussing their recommendations until a consensus is reached. When revisions are invited, a fact-based synthesis statement explaining their decision and outlining what is needed to prepare a revision will be listed below. The following reviewer(s) agreed to reveal their identity: NONE.

Thank you for telling your neuroscience outreach storey.
